# Evaluating the operational efficiency of the compact urban medical group in Qiqihar based on the three-stage DEA and Malmquist index model

**DOI:** 10.3389/fpubh.2025.1636769

**Published:** 2025-08-18

**Authors:** Ye Xing, Chong Tian, Jiayu Wang, Yanting Liu, Jun Tao, Taoyu Lin, Yan Zhou, Yue Wang, Xue Bai

**Affiliations:** ^1^School of Nursing, Tongji Medical College, Huazhong University of Science and Technology, Wuhan, China; ^2^Public Health Department, Wuhan Pulmonary Hospital, Wuhan, China; ^3^Nursing Department, The People’s Hospital of Suzhou New District, Suzhou, China; ^4^Zhuhai People’s Hospital (The Affiliated Hospital of Beijing Institute of Technology, Zhuhai Clinical Medical College of Jinan University), Zhuhai, China; ^5^Institute for Hospital Management of Henan Province, Zhengzhou, China

**Keywords:** DEA, Malmquist, urban medical group, efficiency, influencing factors

## Abstract

**Background:**

Since the establishment of medical alliances, a key issue regarding their ability to better address the imbalance in China’s medical resources lies in the changes in operational efficiency before and after their formation. This study focuses on urban medical groups, a reform model of medical alliances, and systematically analyzes the changes in operational efficiency before and after the group-based reform, aiming to provide empirical evidence for improving the group-based management model.

**Methods:**

This study employs a dual-method framework combining three-stage DEA for static efficiency evaluation and Malmquist index analysis for dynamic assessment. Data from 14 medical institutions inform the analysis, utilizing three carefully selected input and three output variables to comprehensively evaluate resource allocation patterns within the medical consortium.

**Results:**

The first-stage DEA evaluation of Qiqihar’s 14 medical institutions reveals baseline efficiency scores with comprehensive efficiency at 0.839, pure technical efficiency at 0.950, and scale efficiency at 0.882. SFA regression identifies regional GDP as positively influencing operational performance while population density and fiscal appropriations demonstrate negative effects. After adjusting for environmental variables and random disturbances in the third-stage analysis, the recalculated efficiency metrics show significant changes. The adjusted comprehensive efficiency declines to 0.774, reflecting more accurate performance measurement after accounting for external factors. Meanwhile, pure technical efficiency improves to 0.971, suggesting strong managerial performance when isolated from environmental constraints. Scale efficiency decreases to 0.800, indicating suboptimal operational size remains a persistent challenge.

**Conclusion:**

Medical institution planning must carefully consider local healthcare resource distribution, economic conditions, population characteristics, and varying medical needs to determine appropriate operational scales and infrastructure development. Health authorities should enhance coordination among medical groups by breaking institutional barriers and promoting resource sharing to create synergistic collaborations that improve overall service quality and efficiency. Continuous infrastructure improvements remain essential for meeting evolving public healthcare demands while maintaining optimal service delivery standards.

## 1 Introduction

China has long been confronted with the polarization effect of medical and health resources. The unbalanced development problem of “the big getting bigger and the small getting smaller” severely restricts the overall effectiveness of the medical service system (1, 2). To address this predicament, China has established the Medical Consortium system (also known as “medical alliances”) (3). Through cross-level and cross-regional resource integration, efforts are made to improve service quality and cost-control capabilities. Among the four major medical consortium models currently formed, the urban medical group, as an integrated medical service organization with the most distinctive Chinese characteristics, its grid-based layout and system-remodeling practice have important exemplary significance for optimizing regional resource allocation (4).

The establishment of urban medical groups takes full account of the distribution characteristics of urban medical resources. Through a three-level collaborative network of tertiary hospitals, secondary hospitals, and community health service institutions, efforts are made to promote the formation of a new pattern of hierarchical diagnosis and treatment (5). According to differences in the degree of collaboration, they can be divided into four types: compact, semi-compact, loose, and composite (6). This diversified organizational form not only adapts to the economic development levels and resource allocation situations in different regions but also provides an institutional foundation for the generation of synergy effects.

By systematically reviewing the application of Data Envelopment Analysis (DEA) in medical efficiency research, existing achievements present a multi-dimensional and multi-level analytical framework. At the international macro-level, DEA is widely used in the efficiency evaluation and comparative study of medical systems among countries (7). For example, Top M applies DEA to a cross-national comparative study of the healthcare systems of 36 African countries, revealing significant differences in the efficiency of medical resource allocation among different countries (8). At the regional meso-level, the research focus shifts to the analysis of spatial heterogeneity within a single country (9). For example, Ngobeni’s empirical study on the nine provinces of South Africa shows that the DEA model can effectively identify the gradient differences in the technical efficiency of provincial medical systems (10). At the micro-level, research focuses on the heterogeneous characteristics of medical and health institutions. For example, Pirani’s DEA analysis of general hospitals, specialized hospitals, and multi-specialized hospitals in southwestern Iran reveals the differential performance of the operational efficiency of different types of medical institutions (11). Nunes AM applies a network data envelopment analysis approach to conduct a comparative study on the operational efficiency of Portuguese public hospitals before and after the COVID-19 pandemic, systematically evaluating the changing characteristics of resource allocation efficiency and service outputs in medical institutions amid the pandemic impact from an input–output perspective (12). The research team led by Ferreira DC uses data envelopment analysis techniques to carry out efficiency measurement studies on public healthcare institutions within Portugal’s National Health Service. From an input–output perspective, the research systematically explores the relative technical efficiency levels of hospital operations (13).

It is worth noting that Chinese scholars make good progress in the localization innovation and multi-level application of the DEA method. At the national governance level, Gong introduces the network DEA model and uses the network DEA method to evaluate the overall efficiency and the efficiency of two sub-stages of the medical systems in various provinces of China after the implementation of the medical reform (14). At the regional coordination level, Du utilizes the DEA model to deeply explore the correlation between quality and efficiency at the national overall level and in the eastern, central, and western regional groups (15). At the institutional operation level, Jing, by using the MaxDEA analysis tool, conducts a comparative analysis of the efficiency differences between public and private hospitals in Beijing, China (16). However, the existing literature has not fully focused on the impact mechanism of medical group reform on operational efficiency. Especially in the construction of close-knit urban medical groups, there is a lack of systematic efficiency assessment research on whether their efficiency has improved or deteriorated compared to before the formation. This theoretical gap restricts the deepening and advancement of medical group reform.

Current DEA applications in healthcare efficiency research exhibit spatial limitations, particularly in assessing urban medical groups—the core of medical consortium reforms. Existing evaluation systems remain constrained to traditional organizational forms, failing to capture the dynamic resource-sharing networks within medical groups. This methodological gap creates two deficiencies: (1) inability to measure internal resource interaction efficiency, and (2) lack of quantitative evidence for policy evaluation.

Qiqihar City’s 2023 national pilot of compact urban medical groups demonstrates progress through grid-based management and regional medical center coordination. This study analyzes pre/post-reform efficiency changes among member institutions, identifying drivers of performance variation. The findings offer empirical support for optimizing resource allocation and improving consortium management, with direct implications for public hospital reform and hierarchical diagnosis-treatment systems.

## 2 Materials and methods

### 2.1 Data and variables

#### 2.1.1 Data sources

This study examines three representative compact urban medical groups in Qiqihar City (First Hospital, Traditional Chinese Medicine Hospital, and First Affiliated Hospital of Medical College) employing a “1 + N + N” collaborative model. This framework integrates core hospitals with secondary and community healthcare institutions through vertical resource integration, establishing a three-tiered grid-based service network.

In April 2024, structured questionnaires are administered to stakeholders (health administrators, core hospitals, and member institutions) to collect objective operational data on organizational structure, mechanisms, and resource allocation. Given that the aforementioned research subjects completed the establishment of medical groups in 2023, data from different years before and after their formation were collected to further enhance the scientific rigor and comprehensiveness of the comparative analysis. The structured questionnaire adopts a “dual-channel verification” approach, synchronously collecting institutional annual reports and statistical ledgers from health commissions to ensure data consistency. The design of the questionnaire has been reviewed by multiple healthcare management experts, with its content covering core indicators of input, output, and environmental variables, thus guaranteeing data quality. The survey focuses exclusively on institutional characteristics, avoiding personal data requiring ethical review. This approach provides empirical insights into medical consortium development while maintaining research rigor.

#### 2.1.2 Selection of input and output indicators

The Donabedian model focuses on three progressive dimensions of healthcare service quality: structure, process, and outcome, providing a theoretical basis for the “input–output-environmental variables” framework in three-stage Data Envelopment Analysis. The structural dimension, as the “input foundation” of healthcare services, can be defined as the input variables in DEA. The process dimension, acting as the “intermediate transformation link,” can be identified as the environmental variables influencing efficiency. The outcome dimension, serving as the “ultimate goal” of services, is directly defined as the output variables in DEA. This study employs a rigorous three-input/three-output DEA model following completeness, comparability, and data availability principles (17). Input variables encompass: (1) the number of health workers (human resources) (18–20), (2) the number of opening beds (hardware capacity) (21, 22), and (3) the medical business expenditure (financial investment) (23, 24). Output variables include: (1) the number of outpatient cases (18, 22, 23) (2) the number of inpatient cases (service volume) (25–27) and (3) medical business income (economic sustainability) (17, 28, 29).

Pearson correlation analysis (SPSS 26.0) confirmed statistically significant input–output relationships (*p* < 0.01), validating variable selection. This framework enables precise efficiency measurement while capturing medical groups’ multidimensional performance, balancing service delivery with financial viability. The methodology provides robust empirical foundations for identifying operational efficiency determinants in healthcare consortia (Table 1).

**Table 1:** Pearson correlation test for input and output variables.

Variables	Number of health workers	Number of opening beds	Medical business expenditure	Number of outpatient cases	Number of inpatient cases	Medical business income
Number of health workers	1	–	–	–	–	–
Number of opening beds	0.992^**^	1	–	–	–	–
Medical business expenditure	0.976^**^	0.976^**^	1	–	–	–
Number of outpatient cases	0.990^**^	0.973^**^	0.940^**^	1	–	–
Number of inpatient cases	0.996^**^	0.993^**^	0.989^**^	0.979^**^	1	–
Medical business income	0.976^**^	0.975^**^	1.000^**^	0.940^**^	0.989^**^	1

^**^ indicates *p* < 0.01.

#### 2.1.3 Selection of environment variables

This study selects three environmental variables for SFA regression based on Simar and Wilson’s separation hypothesis (30, 31): (1) regional GDP (economic scale effects), (2) population density (agglomeration effects), and (3) fiscal appropriation income (government intervention impact) (22, 32–34). These exogenous factors, while beyond managerial control, influence the input–output efficiency frontier. The SFA analysis isolates environmental influences, enabling precise assessment of managerial efficiency and technical gaps in decision-making units.

### 2.2 DEA methods

Developed by Charnes and Cooper, data envelopment analysis (DEA) is a non-parametric method that evaluates the relative efficiency of decision-making units (DMUs) with multiple inputs and outputs (35, 36). By constructing a production frontier, DEA measures efficiency through the distance between observed values and this frontier, enabling comparative assessment of organizational performance (37).

The DEA system comprises two fundamental models (38, 39). The CCR model assumes constant returns to scale (CRS), measuring overall technical and scale efficiency (40). The BCC model incorporates variable returns to scale (VRS), decomposing efficiency into pure technical efficiency (reflecting production technology) and scale efficiency (assessing input–output proportionality) (41, 42). A scale efficiency value of 1 indicates CRS with optimal proportionality, while values below 1 suggest either increasing or decreasing returns to scale, signaling potential for improvement through scale adjustment (43).

DEA models may be input-oriented (minimizing inputs for given outputs) or output-oriented (maximizing outputs from fixed inputs) (44, 45). This methodological flexibility allows tailored efficiency analysis across different healthcare contexts, making DEA particularly valuable for evaluating complex systems like medical consortia where traditional metrics may be inadequate.

#### 2.2.1 A three-stage DEA model

The three-stage DEA model (Fried et al.) enhances traditional DEA by incorporating stochastic frontier analysis (SFA) (46). This approach first calculates initial efficiency, then uses SFA regression to decompose environmental effects, managerial inefficiency, and random noise while adjusting input–output data, before finally reassessing efficiency with purified data to isolate true managerial performance (1, 47, 48).

**Figure 1:**
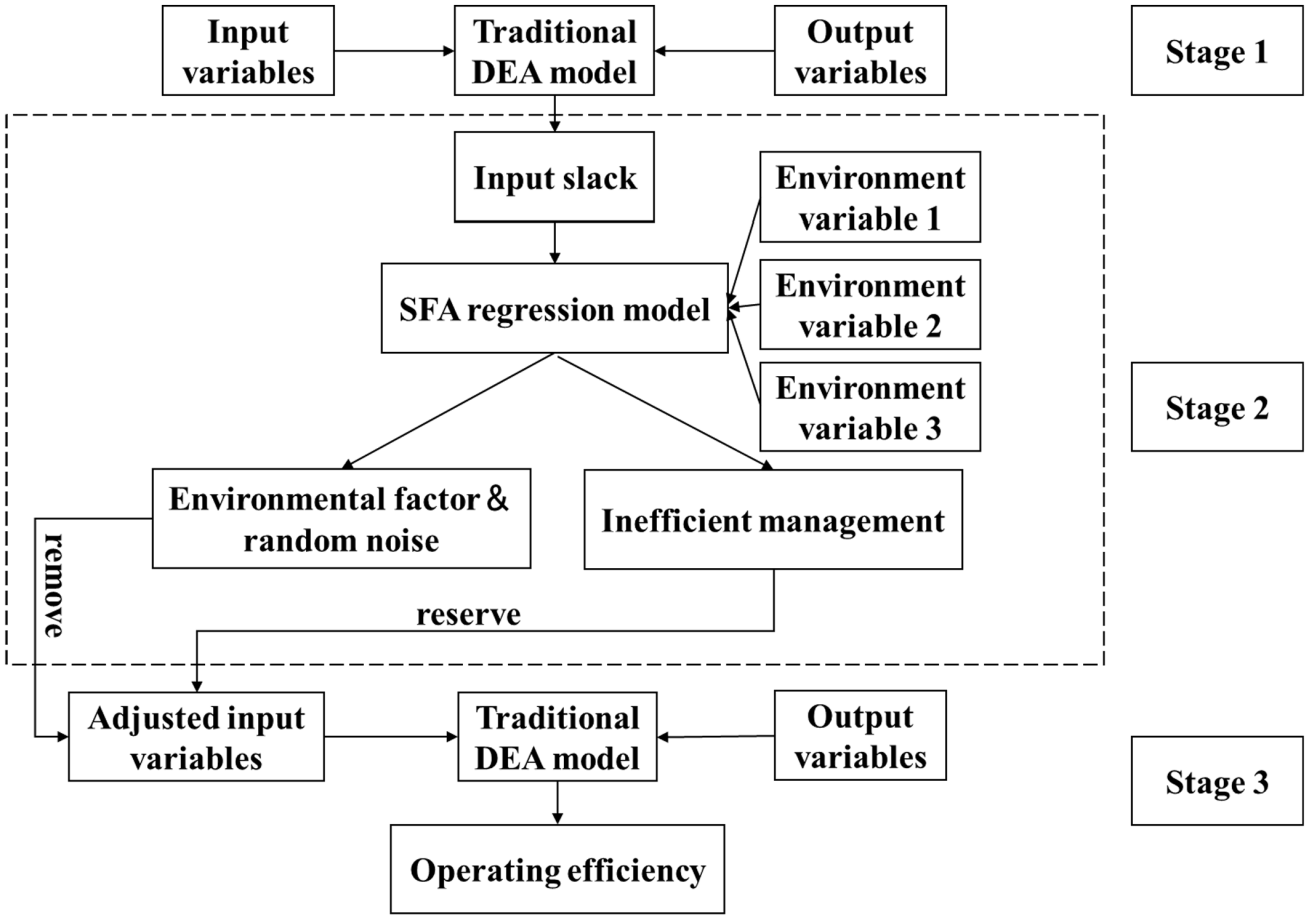
The three-stage DEA model.

The Figure 1 shows the schematic diagram of the three-stage DEA model.

##### 2.2.1.1 Stage 1: traditional DEA model

Considering that the allocation of medical and health resources features dynamic adjustment and the input variables, as basic decision-making parameters, are highly controllable, and also taking into account the public-welfare social responsibilities of medical institutions, this study selects the input-oriented BCC model to calculate the initial efficiency.

Suppose there are *n* DMUs. Each *DMU_j_* has *m* input factors (i=1,2,⋯,m ) and *k* output factors (r=1,2,⋯,k). Then the model is:

(1) $$\min[\theta_{j}-\varepsilon (\sum_{i=1}^{m}s_{\mathrm{ij}}^{-})+\sum_{r=1}^{k}s_{\mathrm{rj}}^{+}]$$

(2) $$s_{.}t_{.}\{\begin{array}{c} \sum_{j=1}^{n}\lambda_{j}x_{\mathrm{ij}}=\theta_{j}x_{\mathrm{ij}}-s_{\mathrm{ij}}^{-},i=1,2,\cdots ,m\\ \sum_{j=1}^{n}\lambda_{j}y_{\mathrm{rj}}=y_{\mathrm{rj}}+s_{\mathrm{ij}}^{+},r=1,2,\cdots ,k\;\;\;\\ \sum_{j=1}^{n}\lambda_{j}=1\;\\ \lambda_{j}\geq 0,s_{\text{in}}^{-}\geq 0,s_{\mathrm{rj}}^{+}\geq 0,j=1,2,\cdots ,n \end{array}\}$$

$\theta_{j}$ is the technical efficiency value of the j-th DMU. $s_{\mathrm{ij}}^{-}$ and $s_{\mathrm{rj}}^{+}$ are the input and output slack variables respectively, and ε is the non-Archimedean infinitesimal. $x_{\mathrm{ij}}$ and $y_{\mathrm{rj}}$ are the input factors and output factors respectively, and $\lambda_{j}$ represents the weight.

The comprehensive technical efficiency (*TE*) measured by the BCC model can be decomposed into the product of pure technical efficiency (*PTE*) and scale efficiency (*SE*), that is, TE = PTE * SE (42, 49, 50).

##### 2.2.1.2 Stage 2: SFA model

Developed by Aigner, SFA is a parametric method that estimates production frontiers while accounting for random disturbances (51). Its key innovation lies in decomposing deviations into: (1) random statistical noise and (2) managerial inefficiency (52). This separation enables more accurate efficiency evaluation of decision-making units by isolating true performance from stochastic variability.

To eliminate the influence of environmental factors and random interference on efficiency, the SFA model is used to decompose the input slack variable $s_{\mathrm{ij}}^{-}$. The SFA model is as follows:

(3) $$s_{\mathrm{ij}}^{-}=f_{i}(Z_{j};\beta_{i})+\nu_{\mathrm{ij}}+u_{\mathrm{ij}}$$

$Z_{j}$ is the environmental variable, $\beta_{i}$ is its coefficient, $v_{\mathrm{ij}}$ is the random interference term, and $v_{\mathrm{ij}}$~$N(0,\sigma_{\mathrm{vi}}^{2})$; $u_{\mathrm{ij}}$ is the management noise, that is, management inefficiency, which follows a truncated normal distribution $u_{\mathrm{ij}}\sim N^{+}$($u_{i},\sigma_{\mathrm{ui}}^{2}$); $v_{\mathrm{ij}}$ and $u_{\mathrm{ij}}$ are independent and uncorrelated.

Separate the random interference term and management inefficiency, and use the adjustment formula to adjust all DMUs to the same external environment. The adjustment formula is:


(4) $$x_{\mathrm{ij}}^{*}=x_{\mathrm{ij}}+[\max(f(Z_{j};{\hat{\beta }}_{i}))-f(Z_{j};{\hat{\beta }}_{i})]-[\max(\nu_{\mathrm{ij}})-\nu_{\mathrm{ij}}]$$

$x_{\mathrm{ij}}^{*}$ represents the input value of the i-th input factor of the j-th decision-making unit after adjustment; $x_{\mathrm{ij}}$ is the input amount of the decision-making unit before adjustment; $\left[\max(f(Z_{j};{\hat{\beta }}_{i}))-f(Z_{j};{\hat{\beta }}_{i})\right]$ represents the input amount adjusted when the DMU is adjusted to the same external environment; $\left[\max(v_{\mathrm{ij}})-v_{\mathrm{ij}}\;\right]$ represents the input amount adjusted when the DMU is adjusted to the same random error term.

##### 2.2.1.3 Stage 3: adjusted BCC model

This stage removes environmental and stochastic influences through standardized input adjustments (based on second-stage SFA results) while maintaining original outputs. Recalculating efficiency using the adjusted inputs in the BCC model isolates true managerial performance, yielding robust operational efficiency measures for evidence-based decision-making.

#### 2.2.2 Malmquist index model

The Malmquist index, developed from Malmquist’s work, extends DEA methodology to assess total factor productivity changes over time (53). Unlike static three-stage DEA, it constructs intertemporal frontiers enabling longitudinal efficiency comparisons (54). This study innovatively applies this model to analyze pre/post-reform operational efficiency changes in urban medical groups, systematically evaluating reform impacts through dynamic productivity decomposition (technical efficiency change and technological progress components).


(5) $$\begin{array}{l} \text{TFPch}=\text{Effch}\times \text{Techch}=\\ \frac{D_{\mathrm{cj}}^{t+1}(x_{i}^{t+1},y_{r}^{t+1})}{D_{\mathrm{cj}}^{t}(x_{i}^{t},y_{r}^{t})}\times \sqrt{\frac{D_{\mathrm{cj}}^{t}(x_{i}^{t+1},y_{r}^{t+1})}{D_{\mathrm{cj}}^{t+1}(x_{i}^{t}+1,y_{r}^{t+1})}\times \frac{D_{\mathrm{cj}}^{t}(x_{i}^{t},y_{r}^{t})}{D_{\mathrm{cj}}^{t+1}(x_{i}^{t},y_{r}^{t})}} \end{array}$$

(6) $$\text{Effch}=\frac{D_{j}^{t+1}(x_{i}^{t+1},y_{r}^{t+1})}{D_{j}^{t}(x_{i}^{t},y_{r}^{t})}$$

(7) $$\text{Techch}=\sqrt{\frac{D_{j}^{t}(x_{i}^{t+1},y_{r}^{t+1})}{D_{j}^{t+1}(x_{i}^{t+1},y_{r}^{t+1})}\times \frac{D_{j}^{t}(x_{i}^{t},y_{r}^{t})}{D_{j}^{t+1}(x_{i}^{t},y_{r}^{t})}}$$

(8) $$\begin{array}{l} \text{Effch}=\text{Pech}\times \text{Sech}=\\ \frac{D_{\mathrm{vj}}^{t+1}(x_{i}^{t+1},y_{r}^{t+1})}{D_{\mathrm{vj}}^{t}(x_{i}^{t},y_{r}^{t})}\times [\frac{D_{\mathrm{vj}}^{t}(x_{i}^{t},y_{r}^{t})}{D_{\mathrm{vj}}^{t+1}(x_{i}^{t},y_{r}^{t})}/\frac{D_{\mathrm{vj}}^{t}(x_{i}^{t+1},y_{r}^{t+1})}{D_{\mathrm{vj}}^{t+1}(x_{i}^{t+1},y_{r}^{t+1})}] \end{array}$$

$x_{i}^{t}$ and $y_{r}^{t}$ represent the i-th input factor and the r-th output factor in period t respectively. $D_{\mathrm{vj}}^{t}(x_{i}^{t},y_{r}^{t})$ denotes the distance function of *DMU_j_* in period t under the condition of variable returns to scale, and $D_{\mathrm{cj}}^{t}(x_{i}^{t},y_{r}^{t})$ represents the distance function of *DMU_j_* in period t under the condition of constant returns to scale. *TFPch* is the total factor productivity change index, which can be decomposed into the technical efficiency change index (*Effch*) and the technological progress index (*Techch*). The technical efficiency change index can be further decomposed into the pure technical efficiency change index (*Pech*) and the scale efficiency change index (*Sech*).

### 2.3 DEA statistical software

This study employs Pearson correlation tests on input–output variables using SPSS 26.0, followed by three-stage DEA (DEAP 2.1) and Malmquist index (Frontier 4.1) analyses to assess both static and dynamic operational efficiency of DMUs, providing robust quantitative evidence for healthcare reform evaluation.

## 3 Results

### 3.1 Descriptive analysis

A survey of 14 medical institutions revealed average operational metrics per facility: 518 healthcare workers, 545 opening beds, and 256 million yuan in medical expenditures. Service outputs remained stable with 206,300 outpatient cases and 18,928 inpatient cases, while generating 680,000 yuan in medical business income (Table 2). These findings demonstrate balanced development across resource allocation, service delivery, and operational efficiency within the medical group system.

**Table 2:** Descriptive statistics for inputs, outputs, and environment variables.

Variables	Max	Min	Mean	SD
Input variables				
Number of health workers	3,272	21	518	997.590
Number of opening beds	3,366	11	544.786	990.810
Medical business expenditure (10,000 RMB)	216222.4	114	25629.784	60484.338
Output variables				
Number of outpatient cases	1,162,866	3,300	206300.214	387710.835
Number of inpatient cases	133,522	1	18927.786	39376.528
Medical business income (10,000 RMB)	229863.53	68.54	27031.743	64391.332
Environment variable				
Regional GDP (billion RMB)	13.796	3.467	9.344	3.579
Population density (persons/km^2^)	2658.2	75.85	1219.989	1066.212
Fiscal appropriation income (10,000 RMB)	9,449	80.26	1772.992	2832.367

### 3.2 Static measurement of operational efficiency of medical groups in compact cities

#### 3.2.1 Results of first-stage SFA model

The 2023 DEA evaluation of three compact urban medical groups in Qiqihar revealed an average comprehensive efficiency of 0.839 across 14 institutions, with pure technical efficiency at 0.950 and scale efficiency at 0.882. Six institutions achieved optimal performance with comprehensive efficiency scores of 1.0, operating on the efficiency frontier. The remaining institutions demonstrated inefficiencies, with either pure technical or scale efficiency below 1.0, indicating potential areas for improvement in resource allocation structures or operational scale optimization. These findings highlight both high-performing facilities and opportunities for system-wide enhancements in healthcare delivery efficiency (Table 3).

**Table 3:** Operational efficiency of Qiqihar compact urban medical group in 2023.

DMU	Stage 1				Stage 3			
	TE	PTE	SE		TE	PTE	SE	
1	0.872	1.000	0.872	drs	0.873	1.000	0.873	drs
2	0.570	0.616	0.926	irs	0.572	0.623	0.918	irs
3	0.580	0.983	0.591	irs	0.419	0.977	0.429	irs
4	1.000	1.000	1.000	–	0.706	1.000	0.706	irs
5	1.000	1.000	1.000	–	1.000	1.000	1.000	–
6	0.639	1.000	0.639	irs	0.618	1.000	0.618	irs
7	0.990	1.000	0.990	drs	0.993	1.000	0.993	drs
8	0.836	1.000	0.836	irs	0.837	1.000	0.837	irs
9	0.540	0.698	0.774	irs	0.503	1.000	0.503	irs
10	1.000	1.000	1.000	–	1.000	1.000	1.000	–
11	0.722	1.000	0.722	irs	0.650	1.000	0.650	irs
12	1.000	1.000	1.000	–	1.000	1.000	1.000	–
13	1.000	1.000	1.000	–	0.671	1.000	0.671	irs
14	1.000	1.000	1.000	–	1.000	1.000	1.000	–
Mean	0.839	0.950	0.882		0.774	0.971	0.800	

drs indicates decreasing returns to scale, irs indicates increasing returns to scale, and – indicates that returns to scale remain unchanged.

#### 3.2.2 Results of second-stage adjusted DEA-BCC analysis

This study employed stochastic frontier analysis (SFA) to examine input slack variables against standardized environmental factors. All input variables demonstrated statistically significant LR statistics (α = 0.05) with γ values approaching 1, confirming that operational efficiency variations primarily reflect management inefficiency rather than random noise. These findings validate the importance of environmental adjustments in healthcare efficiency evaluations.

Regression analysis revealed regional GDP negatively correlated with input slack, suggesting economic development enhances efficiency by reducing resource redundancy. Conversely, population density and fiscal appropriations showed positive associations, indicating population aggregation may worsen resource misallocation while increased subsidies potentially diminish allocation efficiency. These results demonstrate distinct regional economic and policy impacts on medical resource utilization (Table 4).

**Table 4:** SFA model regression results.

Variables	Number of health workers slack	Number of opening beds slack	Number of medical business expenditure slack
Constant	−10.449^**^	−6.214^**^	83.505
Regional GDP	−480.862^**^	−670.100^**^	−3355.476
Population density	310.867^**^	380.471^**^	595.936
Fiscal appropriation income	31.579^**^	37.455^**^	352.130
$\sigma^{2}$	2294.656^**^	3704.629^**^	97126.213^**^
γ	1.000^**^	1.000^**^	1.000^**^
LR test of the one-sided error	8.752^*^	8.977^*^	10.679^**^

^*^ indicates *p* < 0.05, ^**^ indicates *p* < 0.01.

#### 3.2.3 Results of THIRD-STAGE DEA analysis

The three-stage SFA-adjusted results reveal an average comprehensive efficiency of 0.774 for Qiqihar’s medical groups, representing a 0.065 decrease from unadjusted values. Six institutions (DMU3,4,6,9,11,13) show notable efficiency declines (0.021–0.329), demonstrating significant environmental influence. These findings confirm that traditional methods overestimate efficiency when failing to account for environmental factors, highlighting the importance of proper adjustment in healthcare performance evaluation.

Four institutions (DMU1-2 included) demonstrate significantly higher efficiency after environmental adjustment, revealing their operational capabilities are previously constrained by unfavorable conditions. This underscores the critical importance of environmental factor correction for objective performance evaluation in healthcare systems.

After adjustment, average pure technical efficiency improves from 0.950 to 0.971, with 12 institutions reaching optimal levels. However, DMU2’s score of 0.971 remains below average, indicating persistent technical deficiencies in core medical capabilities despite environmental adjustments.

Post-adjustment analysis reveals a significant decline in average scale efficiency from 0.882 to 0.800, with only four institutions maintaining optimal performance versus six previously. Six institutions including DMU3 and DMU4 score below average, confirming scale efficiency as the primary constraint on operational improvement in urban medical groups, aligning with Kirigia’s findings on healthcare system performance limitations (49). However, Pirani’s research reveals that hospitals demonstrate favorable performance in terms of scale efficiency, which is not a key impediment to enhancing hospital efficiency (11). These findings collectively highlight the complexity of improving healthcare system efficiency, as reflected in the coexistence of systemic performance limitations and advantages in scale efficiency, which provides important theoretical references and directional guidance for subsequent related research.

Post-adjustment analysis reveals distinct returns-to-scale patterns: eight institutions (including DMU2,3) show increasing returns, suggesting potential benefits from additional resource inputs; DMU1 and DMU7 demonstrate decreasing returns, indicating suboptimal resource utilization requiring reallocation; while four institutions (DMU5,10) maintain constant returns, reflecting ideal input–output proportionality. These findings highlight the need for differentiated resource allocation strategies across medical institutions based on their scale efficiency characteristics, with increasing-return institutions benefiting most from marginal investments and decreasing-return units requiring operational optimization to improve productivity.

#### 3.2.4 Bootstrap-DEA robustness analysis

To address potential sampling variability in small-sample DEA applications, this study employs the Bootstrap-DEA methodology. The resampling technique generates pseudo-samples to establish bias-corrected efficiency estimates with corresponding 95% confidence intervals for each DMU, thereby validating the statistical robustness of efficiency measurements.

Computational results demonstrate that all 14 DMUs’ original efficiency estimates, including TE, PTE, and SE, fall within their respective bootstrap confidence intervals. The interval widths reveal distinct robustness patterns: PTE > TE > SE, with scale efficiency exhibiting greater variability due to inter-DMU heterogeneity.

These findings not only substantiate the hypothesis that scale inefficiency constitutes the primary constraint on overall performance improvement, but more importantly, confirm the statistical reliability of bootstrap-corrected efficiency estimates in small-sample contexts. The methodological approach provides an effective solution for DEA applications with limited observations, particularly in healthcare system evaluations where sample size constraints are common (Table 5).

**Table 5:** Bootstrap-DEA robustness analysis results.

DMU	TE	BTE	95% CIs	PTE	BPTE	95% CIs	SE	BSE	95% CIs
1	0.873	0.862	[0.845, 0.896]	1.000	0.997	[0.990, 1.000]	0.873	0.865	[0.832, 0.905]
2	0.572	0.565	[0.541, 0.598]	0.623	0.619	[0.595, 0.648]	0.918	0.912	[0.885, 0.946]
3	0.419	0.410	[0.390, 0.445]	0.977	0.973	[0.958, 0.992]	0.429	0.421	[0.395, 0.462]
4	0.706	0.696	[0.678, 0.732]	1.000	0.996	[0.989, 1.000]	0.706	0.699	[0.670, 0.745]
5	1.000	0.992	[0.982, 1.000]	1.000	0.998	[0.993, 1.000]	1.000	0.994	[0.980, 1.000]
6	0.618	0.608	[0.589, 0.645]	1.000	0.997	[0.991, 1.000]	0.618	0.610	[0.580, 0.652]
7	0.993	0.986	[0.975, 1.000]	1.000	0.999	[0.996, 1.000]	0.993	0.987	[0.968, 1.000]
8	0.837	0.827	[0.809, 0.859]	1.000	0.996	[0.988, 1.000]	0.837	0.830	[0.800, 0.869]
9	0.503	0.493	[0.475, 0.529]	1.000	0.995	[0.987, 1.000]	0.503	0.495	[0.465, 0.542]
10	1.000	0.990	[0.980, 1.000]	1.000	0.997	[0.992, 1.000]	1.000	0.993	[0.978, 1.000]
11	0.650	0.639	[0.619, 0.679]	1.000	0.996	[0.989, 1.000]	0.650	0.642	[0.608, 0.685]
12	1.000	0.993	[0.985, 1.000]	1.000	0.998	[0.995, 1.000]	1.000	0.995	[0.982, 1.000]
13	0.671	0.661	[0.639, 0.699]	1.000	0.997	[0.990, 1.000]	0.671	0.663	[0.627, 0.708]
14	1.000	0.991	[0.976, 1.000]	1.000	0.999	[0.997, 1.000]	1.000	0.992	[0.975, 1.000]
Mean	0.774	0.770	[0.751, 0.798]	0.971	0.968	[0.956, 0.985]	0.800	0.795	[0.732, 0.867]

BTE, BPTE, and BSE represent the bias-corrected efficiency estimates derived from the Bootstrap-DEA procedure.

### 3.3 Dynamic measurement of operational efficiency of medical groups in compact cities

The Malmquist index analysis demonstrates a 1.160 average total factor productivity improvement in Qiqihar’s compact urban medical groups. Scale efficiency growth (1.068) and technological progress (1.091) drive this enhancement, while pure technical efficiency (0.995) shows minimal negative impact. These findings indicate substantial operational efficiency gains following the groups’ establishment.

The analysis reveals performance disparities among the 14 institutions, with 42.9% (6) showing technical efficiency declines. This includes 14.3% (2) with reduced pure technical efficiency and 35.7% (5) demonstrating scale efficiency deficiencies. While technological progress meets benchmarks in 92.9% of cases (13), 28.6% (4) underperform in total factor productivity, indicating persistent challenges in resource allocation and technology adoption that require targeted interventions (Table 6).

**Table 6:** Changes and decomposition of total factor production efficiency of medical institutions.

DMU	effch	techch	pech	sech	tfpch
1	0.985	1.014	1.000	0.985	0.999
2	0.993	1.341	0.987	1.006	1.332
3	3.139	1.031	1.104	2.842	3.235
4	1.000	0.781	1.000	1.000	0.781
5	1.000	1.364	1.000	1.000	1.364
6	0.802	1.112	1.000	0.802	0.892
7	0.990	1.066	1.000	0.990	1.055
8	0.872	1.105	1.000	0.872	0.963
9	1.061	1.130	0.854	1.242	1.200
10	1.000	1.180	1.000	1.000	1.180
11	0.933	1.108	1.000	0.933	1.034
12	1.000	1.095	1.000	1.000	1.095
13	1.116	1.041	1.000	1.116	1.162
14	1.000	1.033	1.000	1.000	1.033
Mean	1.063	1.091	0.995	1.068	1.160

## 4 Discussion

This study develops a two-dimensional assessment framework combining three-stage DEA and Malmquist index methods. The approach objectively evaluates 2023 operational efficiency in Qiqihar’s medical groups by eliminating environmental biases, while dynamically tracking productivity trends through decomposition of technical efficiency and technological progress drivers. The integrated methodology provides comprehensive static and dynamic efficiency insights.

The analysis reveals an average adjusted comprehensive technical efficiency of 0.774 across Qiqihar’s urban medical groups, indicating substantial operational inefficiencies. Management shortcomings and suboptimal resource utilization lead to significant input–output conversion losses, with resources failing to achieve full optimization potential. Notable inter-institutional variations in efficiency scores highlight critical disparities in three key operational dimensions: (1) resource allocation patterns, (2) personnel deployment strategies, and (3) equipment utilization rates. These structural differences directly contribute to the observed efficiency gaps, suggesting that standardized optimization protocols could yield measurable improvements. The findings identify specific areas requiring intervention to enhance overall system performance while maintaining necessary service quality standards.

The improved efficiency scores after environmental adjustment demonstrate significant environmental and stochastic influences on medical institution performance, consistent with Liu’s findings (55). Regions with stronger economic development particularly benefit from greater medical service demand, which systematically enhances operational efficiency through improved resource utilization and service delivery mechanisms.

The adjusted average pure technical efficiency of 0.971 demonstrates effective resource utilization across most institutions, reflecting successful knowledge transfer from core hospitals. However, one institution’s lower score suggests inadequate or misaligned support, requiring targeted interventions including enhanced needs assessment, improved internal resource management, and strengthened collaborative mechanisms with core hospitals to address specific technical deficiencies.

The adjusted average scale efficiency of 0.800 reveals significant optimization challenges across the medical consortium. Eight institutions demonstrate increasing returns to scale, where additional resource investments yield proportionally greater outputs, suggesting underutilized capacity. Conversely, two institutions show decreasing returns, indicating resource saturation where expanded inputs fail to proportionally increase outputs, necessitating strategic downsizing. Four institutions maintain optimal constant returns, achieving perfect input–output proportionality. These findings highlight the critical need for differentiated resource allocation strategies - expansion for increasing-return institutions, optimization for constant-return facilities, and rationalization for decreasing-return units to maximize system-wide efficiency.

Post-establishment analysis reveals significant technical efficiency gains across the medical consortium, with an average improvement of 1.063 despite 42.9% of institutions showing temporary declines. The net positive trend reflects successful implementation of three key collaborative mechanisms: (1) enhanced resource-sharing platforms, (2) systematic technology-exchange programs, and (3) standardized staff training protocols. These structural interventions enable more effective utilization of existing technologies, translating to measurable service output increases. The findings indicate that during the transition phase, despite the potential fluctuating characteristics of the performance level of individual institutions, the medical consortium model, relying on inter-institutional learning mechanisms, has significantly achieved efficiency improvement in the short term through resource sharing and technical collaboration.

Technological progress analysis shows only one institution scoring below benchmark, confirming successful integration of medical resources through the consortium model. The group’s operational framework effectively disseminates advanced medical technologies across member institutions, driving system-wide technological upgrades that enhance service quality and clinical capabilities.

Within the scope covered by existing data, the compact medical group model, through the collaborative interaction between core hospitals and member institutions, demonstrates a certain positive role in terms of technical efficiency and scale efficiency, with 12 institutions achieving optimal pure technical efficiency and 9 reaching scale efficiency targets. This demonstrates effective group-wide implementation of standardized management protocols, precise technology deployment, and optimized resource scaling that collectively minimize waste while maximizing operational performance.

The average total factor productivity reaches 1.160, with 71.4% of institutions exceeding benchmark performance. While 28.6% show suboptimal results, the combined effects of technical efficiency improvements (1.063) and technological progress (1.091) drive system-wide productivity growth, demonstrating the consortium’s success in enhancing operational performance.

SFA regression demonstrates GDP’s significant negative association with input slack, indicating high-GDP regions achieve superior resource utilization through three mechanisms: (1) greater baseline healthcare investments, (2) advanced equipment availability, and (3) preferential talent acquisition. These economic advantages create systemic efficiency gains that optimize service quality-output ratios.

Medical institutions in high population density areas with substantial fiscal allocations face complex efficiency challenges despite their apparent resource advantages. While these hospitals demonstrate strong staffing levels and bed capacity, many engage in uncontrolled expansion and excessive resource investment without proper needs assessment. This unscientific growth leads to significant resource allocation imbalances, where additional inputs fail to generate proportional service improvements. The resulting inefficiencies reduce overall health resource productivity, creating substantial waste (56). These findings highlight how well-resourced environments can paradoxically encourage suboptimal investment decisions when expansion lacks evidence-based planning and rigorous outcome evaluation frameworks.

Optimal healthcare resource allocation requires balancing availability with utilization efficiency to prevent overinvestment waste. Evidence-based planning ensures resources effectively meet population needs while improving service quality and operational performance (57). This approach enables urban medical groups to achieve sustainable development through three key outcomes enhanced equity in service delivery, systematic allocation optimization, and measurable efficiency gains across the healthcare system.

## 5 Conclusion

Efficient resource allocation in compact urban medical groups significantly influences service quality and population health outcomes. Research demonstrates substantial input–output imbalances across institutions, with regional GDP, population density, and fiscal allocations directly impacting allocation efficiency. The observed inter-institutional variations confirm that healthcare resource distribution operates within a complex socioeconomic ecosystem rather than as an isolated system, requiring integrated planning approaches that account for these environmental determinants.

Resource imbalances across medical institutions create dual challenges of wasteful surpluses and critical shortages that compromise service accessibility and quality. These disparities necessitate tailored regional planning that carefully balances existing resources, economic conditions, population demographics, and healthcare demands to develop institution-specific strategies that maximize system-wide efficiency and service equity.

Compact urban medical groups may pursue a gradual transition from scale expansion to intensive development via three key improvements: standardized management systems, optimized service processes, and enhanced staff competencies. Implementing advanced management approaches and technologies while strengthening internal governance structures enables hospitals to simultaneously elevate operational efficiency, service quality, and technical capabilities without physical expansion. This paradigm shifts from quantity to quality focuses on maximizing existing resource utilization through systematic process refinements and continuous professional development initiatives.

Governments and health authorities may consider implementing five key strategies to help improve the performance of medical groups. First, data-driven resource allocation requires developing long-term plans based on demographic and economic analyses, prioritizing geriatrics and rehabilitation services in aging populations. Second, performance management systems should evaluate institutions using efficiency, quality, and satisfaction metrics, linking results to funding allocations to incentivize improvement. Third, workforce development necessitates targeted training programs through academic partnerships alongside attractive recruitment packages for specialty fields like pediatrics and psychiatry. Forth, digital transformation involves creating unified information platforms enabling data sharing, telemedicine, and appointment systems to streamline services and reduce patient wait times. Fifth, medical alliance integration demands clear institutional roles within networks, standardized referral protocols, and tiered service delivery where advanced hospitals support primary centers through training and technical assistance while community facilities manage routine care and follow-up. These interconnected approaches collectively address resource optimization through scientific planning, performance incentives, human capital investment, technological enablement, and collaborative care models. The strategy balances immediate operational improvements with sustainable capacity building, ensuring both efficiency gains and quality enhancement across the healthcare continuum. Implementation requires coordinated policy support, adequate funding mechanisms, and continuous monitoring systems to adapt to evolving population needs while maintaining service accessibility and clinical standards.

Optimizing health resource allocation and improving urban medical group efficiency are critical for advancing healthcare quality. Coordinated government guidance, management innovation, technological advancement, and resource sharing enable sustainable, high-quality service delivery. This study provides theoretical foundations for compact medical group development while offering practical insights for institutional efficiency evaluation and evidence-based policymaking in healthcare systems.

## 6 Limitations

Efficiency assessment in urban medical groups requires carefully selected variables that align with institutional characteristics while ensuring data availability to measure core competencies. Although quantitative metrics like staffing, infrastructure, and funding reveal basic resource distribution patterns, they fail to capture the multidimensional nature of true operational efficiency, presenting significant limitations in comprehensive performance evaluation.

Intangible elements significantly influence resource allocation effectiveness, with organizational culture shaping staff behaviors and patient satisfaction reflecting service quality. These qualitative factors, though difficult to quantify, fundamentally determine operational efficiency and resource utilization patterns in daily healthcare delivery.

Evaluation frameworks relying exclusively on quantitative metrics risk overlooking critical qualitative dimensions, potentially distorting assessment outcomes. Such approaches may produce misleading efficiency measurements that fail to capture the complete operational reality of urban medical groups, particularly regarding service quality and organizational dynamics.

In conclusion, a robust assessment system must integrate qualitative factors alongside quantitative metrics to provide comprehensive insights for optimizing urban medical groups. This balanced approach enables more accurate performance measurement and informed decision-making for sustainable healthcare development.

## Data Availability

The original contributions presented in the study are included in the article/supplementary material, further inquiries can be directed to the corresponding author.
